# Antimicrobial Activity and Sorption Behavior of Al_2_O_3_/Ag Nanocomposites Produced with the Water Oxidation of Bimetallic Al/Ag Nanoparticles

**DOI:** 10.3390/nano12213888

**Published:** 2022-11-03

**Authors:** Sergey O. Kazantsev, Olga V. Bakina, Aleksandr V. Pervikov, Nikolay G. Rodkevich, Nguyen Hong Quang, Lan Anh Le Thi, Sergei S. Timofeev, Aleksandr S. Lozhkomoev

**Affiliations:** 1 Laboratory of Nanobioengineering, Institute of Strength Physics and Materials Science, Siberian Branch, Russian Academy of Sciences, Pr. Akademicheskii 2/4, 634055 Tomsk, Russia; 2 Laboratory of Physical Chemistry of Ultrafine Materials, Institute of Strength Physics and Materials Science, Siberian Branch, Russian Academy of Sciences, Pr. Akademicheskii 2/4, 634055 Tomsk, Russia; 3 Laboratory Military Medicine and Adaptation, Vietnam-Russia Tropical Center, Institute of Bio-Medicine, Ngia Do, Kau Zai, St. Nguyen Van Huen, 63, Hanoi 11307, Vietnam; 4 Laboratory of Toxicity and Tropical Diseases, Vietnam-Russia Tropical Center, Institute of Bio-Medicine, Ngia Do, Kau Zai, St. Nguyen Van Huen, 63, Hanoi 11307, Vietnam

**Keywords:** aluminum, bimetallic nanoparticles, silver, oxidation, alumina, boehmite, bayerite, composite, antimicrobial activity

## Abstract

The water oxidation of bimetallic Al/Ag nanoparticles has been shown to yield nanoscale structures whose morphology, phase composition and textural characteristics are determined by the synthesis conditions. Flower-like nanoscale structures with silver nanoparticles, with an average size of 17 nm, are formed in water at 60 °C. Under hydrothermal conditions at temperatures of 200 °C and a pressure of 16 MPa, boehmite nanoplatelets with silver nanoparticles, with an average size of 22 nm, are formed. The oxidation of Al/Ag nanoparticles using humid air at 60 °C and 80% relative humidity results in the formation of rod-shaped bayerite nanoparticles and Ag nanoparticles with an average size of 19 nm. The thermal treatment of nanoscale structures obtained at a temperature of 500 °C has been shown to lead to a phase transition into γ-Al_2_O_3_, while maintaining the original morphology, and to a decrease in the average size of the silver nanoparticles to 12 nm and their migration to the surface of nanoscale structures. The migration of silver to the nanoparticle surface influences the formation of a double electric layer of particles, and leads to a shift in the pH of the zero-charge point by approximately one, with the nanostructures acquiring pronounced antimicrobial properties.

## 1. Introduction

Recently, the global community has been concerned about the emergence and spread of new resistant strains of bacteria. As a result, there is an urgent need to create antimicrobial agents effective against antibiotic-resistant strains of bacteria and that do not cause the emergence of resistance [1,2,3,4,5]. One of the widely used antibacterial agents is silver, mainly in the form of colloidal particles [6,7,8,9]. Reducing the size of silver nanoparticles leads to a significant increase in its activity, even at low concentrations [5,6,10,11,12,13]. The antibacterial mechanism of silver is based on the ability of silver cations to penetrate inside bacterial cells and interact with their components (e.g., DNA and RNA) and to change the membrane permeability [14], leading to the arrest of the life processes of the bacteria. It should also be noted that silver is effective against multidrug-resistant strains [15,16,17]. High antibacterial activity has led to the wide use of silver nanoparticles in medicine for the treatment of textiles [18], the creation of dressings [19], antibacterial ointments [20], etc. However, unanchored silver nanoparticles can easily be removed from the carrier material during operation. In addition, another serious issue is the natural tendency of silver nanoparticles to agglomerate, leading to a loss in their biological activity.

Promising materials for antibacterial applications include antibacterial composite adsorbents consisting of silver-doped inert carriers capable of trapping bacteria [21,22,23,24,25]. Metal oxides [22,23], carbon materials [24] and silicon oxide [25] are generally used as carriers. Alumina is the most promising material for this application [21,26,27]. It is characterized by a high specific surface area with excellent sorption properties, it is an inert material with respect to the human body and has good biocompatibility [28].

The possibility of the chemical synthesis of silver-doped alumina nanostructures of different phase compositions from metal salts was considered [29,30,31,32,33,34,35,36,37,38,39]. Various organic initiators and stabilizers were used to ensure a high yield of the reaction products and the fixation of silver nanoparticles on the alumina surface. Nanostructures obtained this way had good antimicrobial activity. One of the main disadvantages of the chemical synthesis is the multiple stages and the formation of undesirable by-products that require additional operations for their removal. In addition, the organic stabilizers used are often themselves toxic.

Bicomponent Al_2_O_3_/Ag nanoparticles obtained through the electrical explosion of wires in an oxygen atmosphere have been shown to display strong antimicrobial activity [40]. However, these nanoparticles have a low specific surface area, which limits their use as sorption–bactericidal agents. Recently, the possibility of the water oxidation of electroexplosive bicomponent Al/Ag nanoparticles containing 15 and 9 at. % Ag resulting in the formation of nanosheet structures possessing a high specific surface area was first shown [41,42]. However, the influence of the oxidation conditions of Al/Ag nanoparticles and the thermal treatment of the oxidation products on the sorption and antibacterial properties of the resulting composites was not considered.

In the present work, the features of the sorption–bactericidal Al_2_O_3_/Ag composite formation depending on the conditions of preparation and modes of heat treatment were studied. The influence of synthetic parameters on the physicochemical characteristics and antibacterial activity of composites was evaluated.

## 2. Materials and Methods

### 2.1. Production of Al/Ag Nanoparticles and Al_2_O_3_/Ag Composites

Bimetallic Al/Ag nanoparticles were obtained through the electrical explosion of twisted wires (EEW) of the corresponding metals in an argon atmosphere. Nanoparticles with an Al/Ag atomic ratio of 91:9 were obtained using the method described previously [43].

Bimetallic Al/Ag nanoparticles were oxidized with water according to three methods: (1) An Al/Ag nanoparticle sample weighing 1.00 g was placed in 100 mL of deionized water preheated to 60 °C and stirred for 1 h to oxidize the Al. The solid formed was filtered through a 0.22 μm MCE membrane filter and dried at 100 °C for 2 h. (2) An Al/Ag nanoparticle sample weighing 1.00 g was placed in a climate chamber (TXB-60, Russia) at 60 °C and 80% relative humidity for 72 h. After that, the reaction products were dried at 100 °C for 2 h. (3) An Al/Ag nanoparticle suspension (1.00 g nanoparticles in 100 mL deionized water) was placed in a Teflon-lined autoclave (Tefic HYD-300, China) and kept at 200 °C for 6 h. The resulting solid was filtered using 0.22 μm MCE membrane filters and dried at 100 °C for 2 h.

Then, the composites obtained were subjected to a heat treatment at 500 °C for 2 h to produce γ-Al_2_O_3_/Ag. As a result, a number of samples were obtained, the description of which is given in Table 1.

### 2.2. Characterization of the Materials under Study

The resulting nanoparticles and composites were characterized using X-ray diffraction (XRD) in a Shimadzu XRD 6000 diffractometer operating with Cu Kα radiation. A qualitative phase analysis of the materials under study was carried out with a powder diffraction file (PDF) database PDF-2, released in 2014. The morphology of the materials was characterized using transmission electron microscopy (JEM-2100, JEOL, Tokyo, Japan). The elemental mapping in the nanoparticles and composites was carried out using an X-Max energy-dispersive spectrometer (Oxford Instruments, Abingdon, GB, UK) integrated with a microscope. Zeta potential measurements were performed using the Zetasizer Nano ZSP instrument (Malvern Instruments Ltd., Malvern, Worcestershire, GB, UK) with the use of Zetasizer software. The surface area and pore structure of the materials were measured with nitrogen adsorption/desorption using a Sorbtometer M (Katakon, Novosibirsk, Russia) automatic analyzer. The Ag^+^ release was determined with a voltammetric stripping analysis using an STA analyzer (TomAnalyt, Tomsk, Russia). The measuring electrode was gold–carbon; the reference electrode was silver chloride.

### 2.3. Antibacterial Activity Assay

*E. coli* ATCC 25922, *St. aureus* ATCC 6538P and *MRSA* ATCC 43300 strains were used for the antibacterial studies [44]. The minimum inhibitory concentration (MIC) was determined with a microdilution assay using 96-well plates (300 µL per well). The final concentration of bacterial cells was 1 × 10^5^ CFU/mL. The bacterial suspension was used for no more than 30 min after dilution to preserve cell viability. The bacterial suspension at a concentration of 10^5^ CFU/mL was placed in a liquid nutrient medium containing nanoparticles at a concentration of 0.1–10 mg/mL. The MIC value was the amount of agent that resulted in a microorganism growth arrest.

The batch mode for the adsorption of bacteria on the Al_2_O_3_/Ag composites was used [45]. A 0.9 wt% NaCl solution (normal saline) was used as the medium to prepare the bacterial suspension. The concentration of bacteria in the solution was 1 × 10^5^ CFU/mL. Al_2_O_3_/Ag composite samples weighing 0.100 ± 0.001 g were added to 10 mL of a MRSA suspension. The mixture was stirred at 200 rpm and 25 °C for 10–60 min. To separate the bacteria that were not adsorbed, 3 mL of 60 wt% sucrose solution was added to the suspension. The resulting mixture was centrifuged for 10 min at 3500 rpm. After that, 100 μL of a supernatant was seeded on Petri dishes with dense Müller–Hinton agar medium (NICF, Russia). Petri dishes were incubated for 18 ± 3 h at 37 ± 1 °C. Then, the removal efficiency of the bacteria from the supernatant was calculated (R, %).

### 2.4. Statistical Analysis

A statistical analysis of the degradation rate was performed with a *t*-test on raw (relative weight loss) data using “Two-Sample *t*-test on Rows” tool in OriginPro 8 software (OriginLab Corporation, Northampton, MA, USA). *p*-values of less than 0.05 were considered as statistically significant.

## 3. Results and Discussion

**Figure 1:**
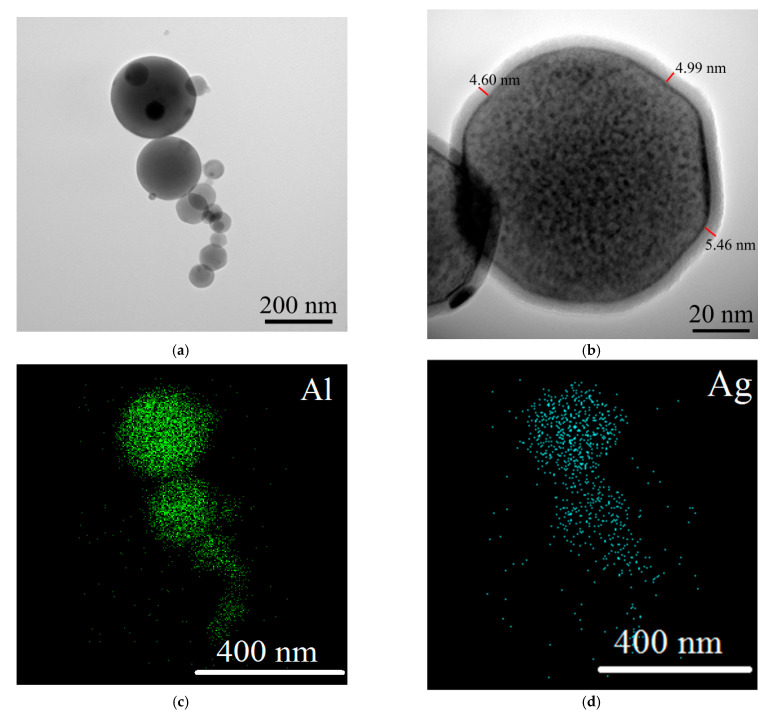
TEM images (**a**,**b**) and TEM-EDS mapping (**c**,**d**) of Al/Ag nanoparticles.

The TEM images (Figure 1a,b) revealed that the Al/Ag nanoparticles were spherical in shape and loosely agglomerated. The TEM-EDS analysis in the mapping mode showed a uniform distribution of Al (91 at. %) and Ag (9 at. %) over the nanoparticle volume (Figure 1c,d). At the same time, the formation of an oxide layer was observed on the nanoparticle surface as a result of powder passivation in air. The layer was not uniform and the average thickness was approximately 5 nm (Figure 1b).

**Figure 2:**
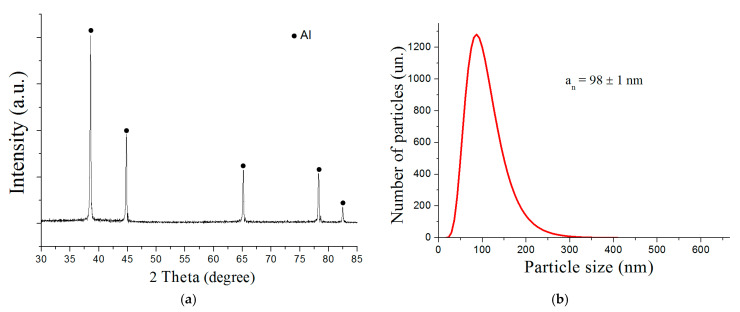
XRD pattern (**a**) and particle size distribution (**b**) of Al/Ag nanoparticles.

According to the XRD phase analysis (Figure 2a), the main peaks in the XRD pattern of the sample obtained corresponded to the Al phase. There were no phases corresponding to silver and the chemical compounds of silver with aluminum on the X-ray diagram. The lattice parameter of aluminum corresponded to the standard value (4.049 Å). This phenomenon may have occurred due to the formation of a supersaturated solid solution based on Al [46] and/or the formation of Guinier–Preston zones in nanoparticles [47] characterized by the extremely small size of the Ag clusters [48,49,50].

The nanoparticles had a log-normal size distribution (Figure 2b) with the average particle size being 98 nm.

**Figure 3:**
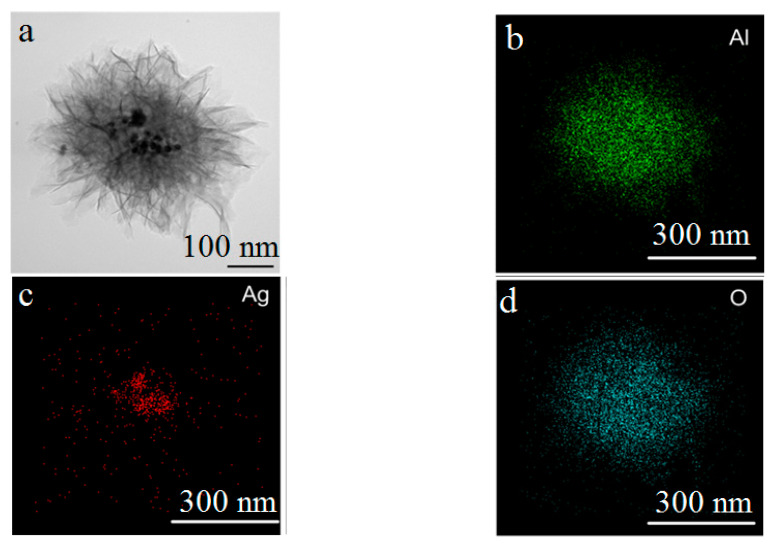
TEM image (**a**) and TEM-EDS mapping (**b**–**d**) of the nanoscale structures obtained through the water oxidation of Al/Ag nanoparticles at 60 °C (sample 1).

The water oxidation of the Al/Ag nanoparticles at 60 °C (sample one) led to the formation of nanosheet agglomerates with sizes up to 1 µm, at the center of which they were enriched with silver nanoparticles with average sizes of approximately 17 nm (Figure 3). Previously, the water oxidation of the EEW aluminum nanoparticles was shown to occur through the dissolution of the metal, resulting in the formation of hollow spheres with the shell of the nanoparticles’ initial oxide layers. Dissolved aluminum species were deposited on the shell in the form of boehmite precursors and arranged in arrays, forming nanosheet structures [51]. In the case of the oxidation of Al/Ag nanoparticles, aluminum was dissolved, releasing silver, which was then assembled into particles unable to move through the initial oxide layer of the Al/Ag nanoparticles.

**Figure 4:**
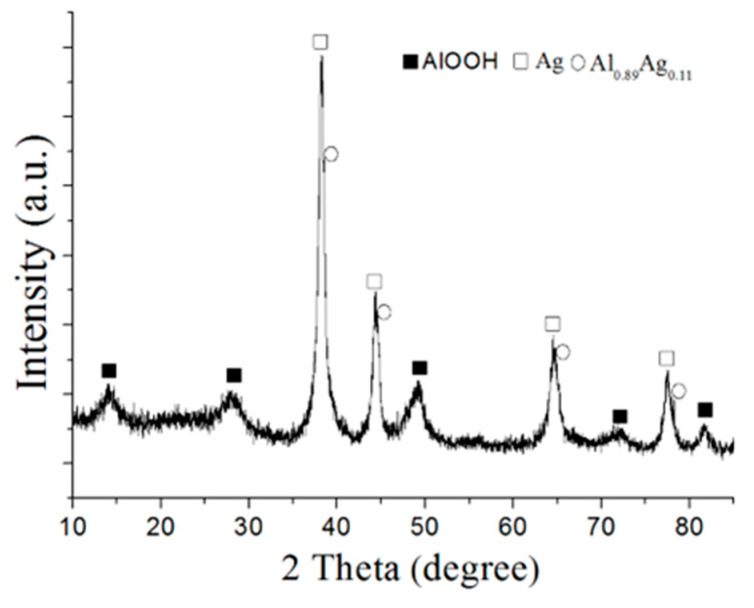
XRD pattern of the nanoscale structures obtained through the water oxidation of Al/Ag nanoparticles at 80% RH at 60 °C (sample 1).

According to the XRD phase analysis (Figure 4), the final water oxidation products were fine crystalline boehmite (AlOOH), as evidenced by the broadened peak in the XRD pattern, silver with the lattice parameter 0.4086 Å (silver FC lattice) and the solid solution phase Al_0.89_Ag_0.11_ with the lattice parameter 0.4053 Å. The peak at 2θ = 38.2° had an asymmetry corresponding to the plane (111). The decomposition of the peak into components revealed two phases: silver and an aluminum-based solid solution. The formation of the solid solution could have been the result of increasing the amount of silver due to the reduction in aluminum content during the reaction as well as the result of increasing the solubility of silver in the aluminum matrix due to an increase in temperature during the reaction or on drying the sample obtained.

**Figure 5:**
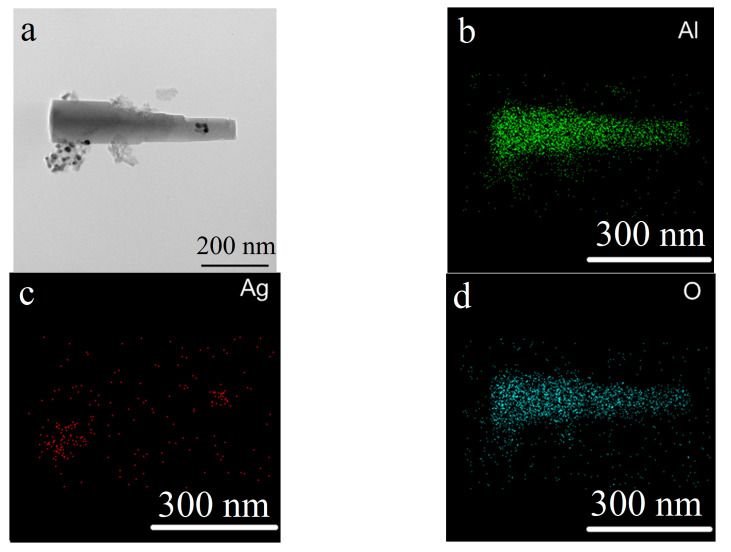
TEM image (**a**) and TEM-EDS mapping (**b**–**d**) of the nanoscale structures obtained through the water oxidation of Al/Ag nanoparticles at 80% RH at 60 °C (sample 2).

The oxidation of the Al/Ag nanoparticles in humid air at 80% RH and a temperature of 60 °C (sample two) yielded rod-shaped aluminum oxide nanoparticles up to 500 nm in length and 100 nm in diameter and spherical silver nanoparticles with an average size of 19 nm (Figure 5). The silver nanoparticles were stabilized with aluminum oxide and were mainly concentrated in agglomerates formed on the dissolution of the aluminum core during the water oxidation.

**Figure 6:**
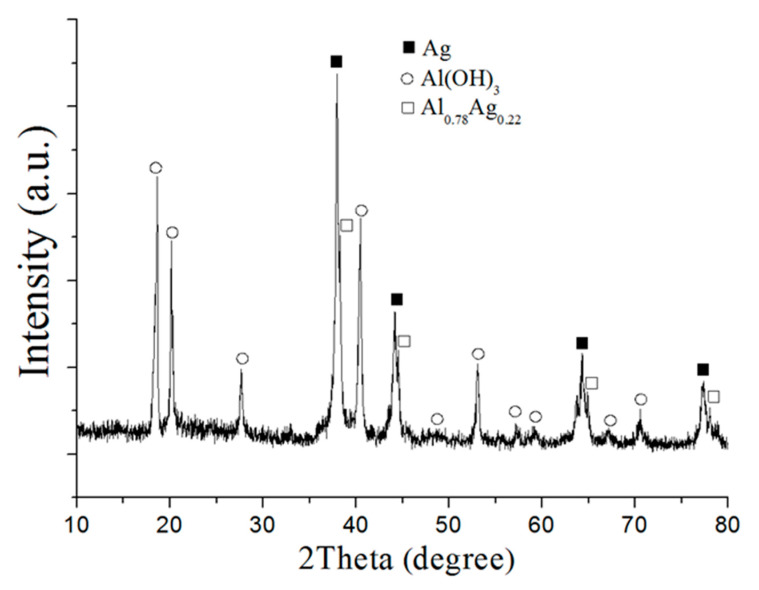
XRD pattern of the nanoscale structures obtained through the water oxidation of Al/Ag nanoparticles at 80% RH at 60 °C (sample 2).

The XRD pattern of the reaction products (Figure 6) indicated the existence of aluminum trihydroxide Al(OH)_3_, silver particles with a standard lattice parameter 4.086 Å and solid solution Al_0.78_Ag_0.22_ with a lattice parameter 4.079 Å.

A lack of water led to a reaction in the external mass diffusion mode through the topochemical mechanism. Increasing the reaction time contributed to the formation of a solid solution with a high silver content, compared with oxidation in excess water. This was most likely because of the diffusion processes being slowed due to a lack of water and a slower oxidation process, which led to the dissolution of silver in the aluminum.

**Figure 7:**
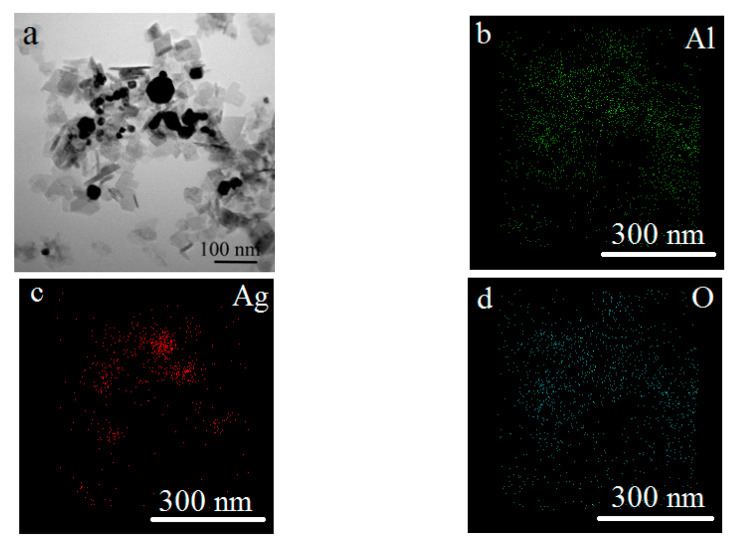
TEM image (**a**) and TEM-EDS mapping (**b**–**d**) of nanoscale structures obtained under HTO (sample 3).

The oxidation of the Al/Ag nanoparticles under hydrothermal conditions (HTO) at 200 °C for 6 h (Sample three) led to the formation of lamellar nanoscale structures up to 100 nm in size and silver-enriched spherical nanoparticles sized 7–100 nm (Figure 7).

**Figure 8:**
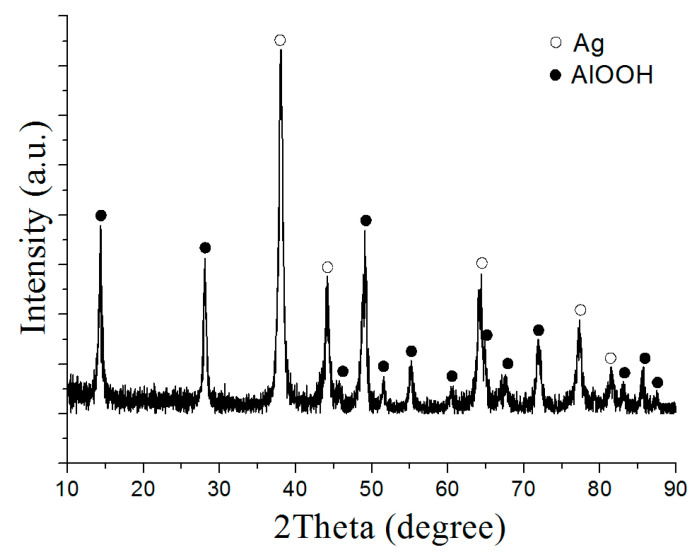
XRD pattern of the nanoscale structures obtained under HTO conditions (sample 3).

The main HTO product of the Al/Ag nanoparticles was boehmite (AlOOH) and silver, with a standard lattice parameter (Figure 8). The formation of solid solutions was not observed, which may have been due to the harsher oxidation conditions. Under HTO conditions, the aluminum oxidized much faster due to the higher temperature, and the solid solution phase did not have time to form. The decomposition of the solid solution due to harsh oxidation conditions was also likely. The high pressure and temperature promoted the formation of a well-crystallized boehmite phase.

**Figure 9:**
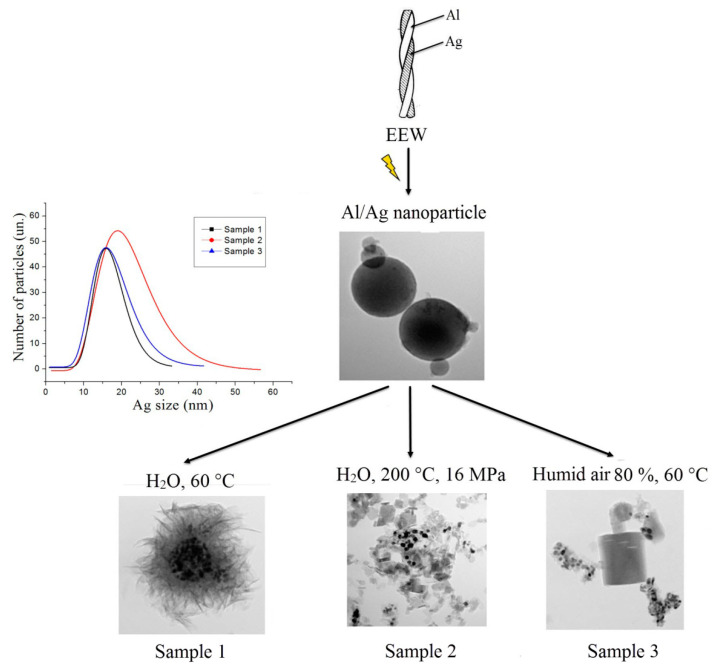
Scheme of different morphology nanostructure formations during water oxidation of Al/Ag nanoparticles under different conditions.

Thus, depending on the conditions of the water oxidation EEW Al/Ag nanoparticles, different ways of nanoscale structure formation could be realized (Figure 9)—the nanoscale structures with the flower-like morphology (nanoflowers) in the center, in which silver nanoparticles with an average size of 17 nm were concentrated (sample one); bayerite nanorods of up to 500 nm with silver particles with an average size of 19 nm (sample two); and boehmite nanoplatelets with silver particles sizes of 22 nm (sample three). The silver particles in the nanoplatelets, in addition to the increased average particle size, were also characterized by a wider size distribution with an increasing particle size.

**Figure 10:**
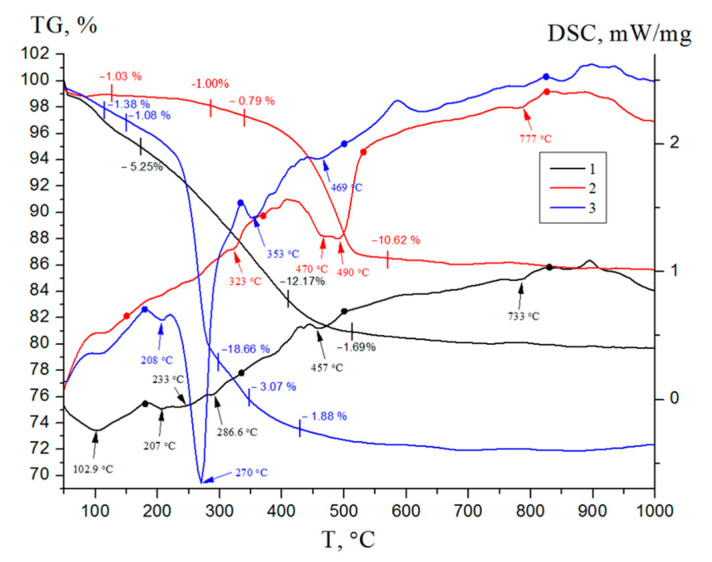
DSC–TG curves of the synthesized nanoscale structures: 1—sample 1; 2—sample 2; 3—sample 3.

The DSC–TG analysis curves (Figure 10) of the nanoscale structures synthesized indicated the process characteristics of nanoscale structures obtained from the EEW Al/AlN nanopowder containing no Ag [52].

**Figure 11:**
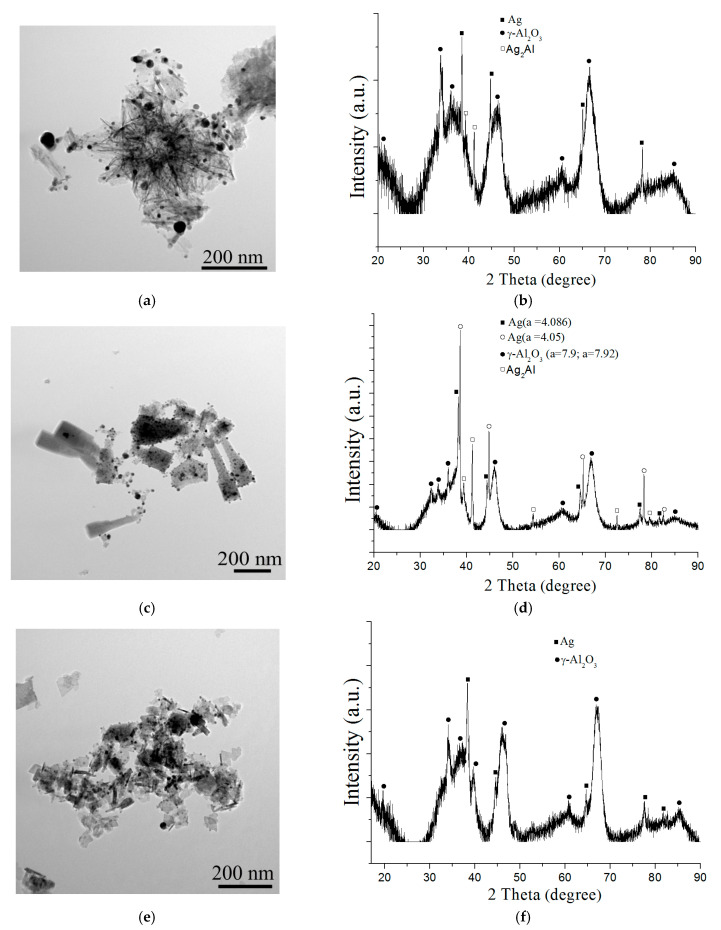
TEM images (**a**,**c**,**e**) and XRD pattern (**b**,**d**,**f**) of the γ-Al_2_O_3_/Ag nanostructures: (**a**,**b**)—sample 4; (**c**,**d**)—sample 5; (**e**,**f**)—sample 6.

All samples were characterized by the loss of adsorbed and structural water after heating to 500 °C, indicating the formation of a more thermodynamically stable γ-Al_2_O_3_ phase. According to the TEM data, the thermal treatment of the nanostructures did not lead to significant changes in the particle morphology, but led to the migration of silver nanoparticles on their surface (Figure 11a,c,e).

In all cases, the formation of γ-Al_2_O_3_ and silver species with a standard lattice parameter of 4.086 Å occurred after the heat treatment at 500 °C (Figure 11b,d,f). The XRD patterns of the flower-like particles (sample four) and nanorods (sample five) showed peaks related to Ag_2_Al intermetallide, which could have formed from the solid solution on heating (Figure 11b,d). Additionally, the formation of a solid solution based on silver, Ag_0.88_Al_0.12_, was observed in the composition of the γ-Al_2_O_3_/Ag nanorods, which could have formed (Figure 11d) on the dissolution of residual aluminum in silver when heating.

**Figure 12:**
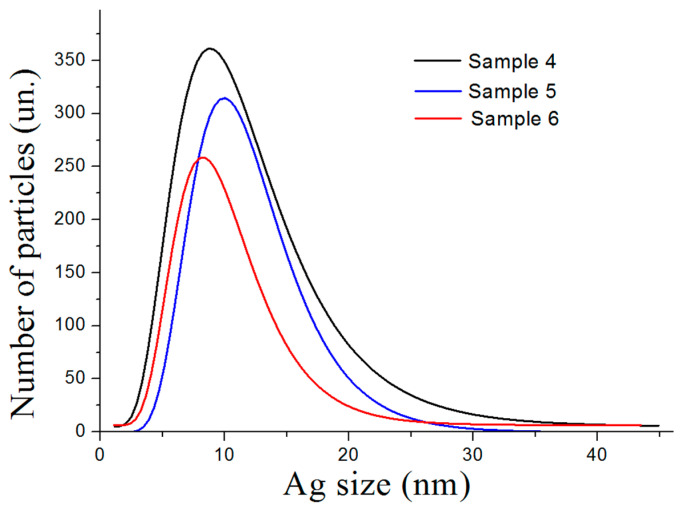
Size distribution of Ag nanoparticles resulted from the thermal treatment of nanostructures at 500 °C.

The Thermal treatment of nanostructures also reduced the average size of the silver nanoparticles to 12 nm (Figure 12). The migration of silver particles and reduction in their size could be related to the thermally induced mobility of silver nanoparticles at temperatures above 200 °C [53].

**Figure 13:**
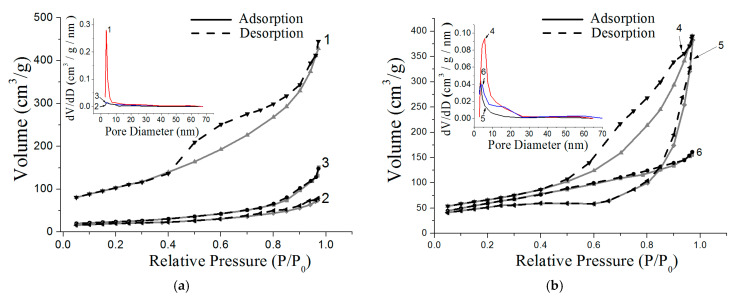
Nitrogen adsorption–desorption isotherms before (**a**) and after (**b**) thermal treatment of nanoscale structures and pore size distribution (inset) for samples 1–6.

The thermal treatment of the synthesized nanoscale structures at 500 °C led to significant changes in the textural characteristics of the samples studied (Figure 13). The appearance of mesopores ranging in size from 5 to 30 nm is a characteristic property of flower-like nanostructures, resulting in a broadening of the peak with a maximum of approximately 5 nm (Figure 13b). At the same time, the sample retained its mesoporous structure and high specific surface area of approximately 300 m^2^/g. The appearance of large mesopores may have been due to the release of silver nanoparticles on the outer surface of the nanosheets from the cavity formed through the dissolution of aluminum. The boehmite platelets and bayerite nanorods did not have a pronounced porous structure. Nitrogen adsorption–desorption curves in the region of relative pressures of 0.1–0.9 were close to being horizontal and practically coincided at all P/P_0_ values (Figure 13a). After the calcination of the Al(OH)_3_/Ag nanorods and AlOOH/Ag nanoplatelets, mesopores appeared in these samples (Figure 13b, inset), which led to an increase in the specific surface area of the nanostructures to 200 and 220 m^2^/g, respectively.

**Figure 14:**
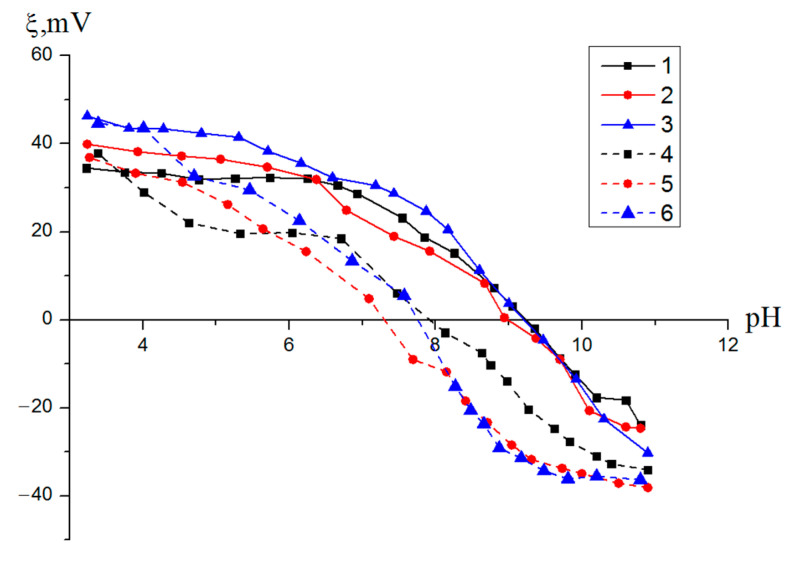
Dependences of the ζ-potential of the nanoscale structures on the pH value for samples 1–6.

The thermal treatment of the nanostructures and the release of silver on the outer surface of the particles contributed to a slight change in their electrokinetic characteristics. There was a shift in the zero-charge point to lower pH values and a general decrease in the ζ-potential in the pH range of 4–7 (Figure 14). Thus, the silver released on the outer surface of the nanoscale structures began to affect the formation of the particle electrical double layer and may have led to a weakening of the electrokinetic trapping of bacteria, with a negative ζ-potential from water.

The antibacterial activity of the nanoscale structures was determined against Gram-negative (*E. coli*) and Gram-positive (*S. aureus*, MRSA) bacteria using a broth microdilution assay. MIC values for the nanoscale structures are shown in Table 2.

Among these nanoscale structures, the lowest MIC values with respect to *E. coli* were observed for sample four (0.16 mg × mL^−1^); the highest MIC values were observed for sample two and sample three (2.5 mg × mL^−1^). Regarding *S. aureus*, the lowest MIC values were observed for sample four and sample five (0.32 mg × mL^−1^); the highest MIC value was observed for sample two (5 mg × mL^−1^). Thus, as for the MIC values, the heat treatment of all the nanoscale structures resulted in an almost 8-fold increase in antibacterial activity against *E.coli* and a 17-fold increase against *S. aureus*. The increase in antibacterial activity of the nanoscale structures after the thermal treatment may have been due to two factors: (1) the migration of the Ag nanoparticles to the periphery of the nanostructures, leading to an increase in the probability of the direct contact of Ag with the bacterial cell membranes; (2) a reduction in the size of silver containing species up to 12 nm.

**Figure 15:**
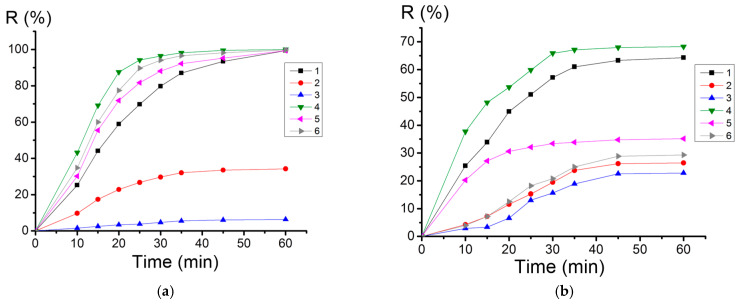
Dependences of the reduction in the bacteria content in the supernatant liquid on the incubation time with nanostructures containing silver (**a**) and without silver (**b**) for samples 1–6. Silver-free samples were of the same morphology, synthesized under the same conditions from Al nanopowder.

It is worth noting that the suspension method of determining the MIC in the nutrient medium was developed for chemotherapeutic drugs in suspension in the ionic form. The low MIC values for the nanostructures could be explained by the small amount of ions released during the exposure. To assess the efficiency of the sorption–bactericidal properties of the nanoscale structures studied, temporal dependences of the reduction in the number of bacteria in the supernatant were obtained (Figure 15a). For comparison, dependences were also obtained for the nanostructures without silver synthesized under similar conditions (Figure 15b).

**Figure 16:**
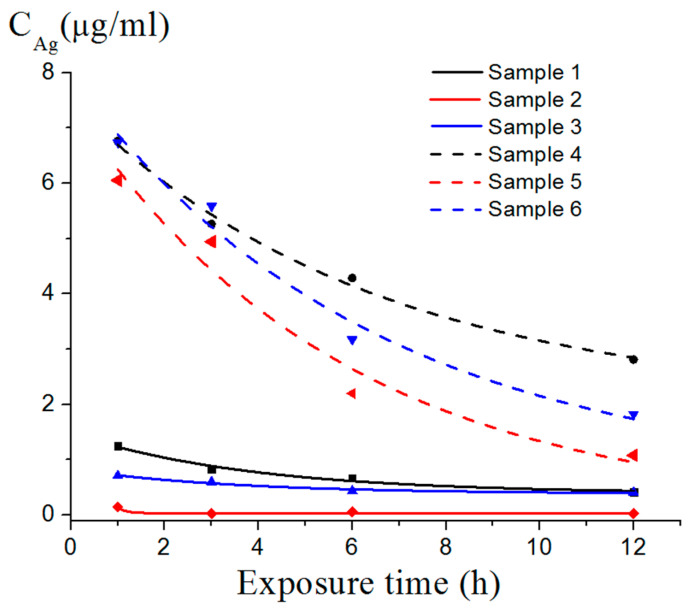
Kinetic curves of silver ion migration.

The curves in Figure 15 show an ambiguous effect of silver depending on the type of nanoscale structure. For nanoplatelets, the contribution of bacterial adsorption leading to a decrease in the number of cells in the supernatant was more pronounced than the effect of silver (Figure 15a,b, curve three), which could have been due to the lower ζ-potential of the Ag nanoplatelets (ζ = 24 mV, pH = 7) and the larger Ag particle size in sample three (Figure 9, inset) compared to other samples. The low ζ-potential prevented the occurrence of an effective interaction with negatively charged bacterial cells, and the large size of the Ag particles ensured a slow release of Ag^+^ ions into the medium, which was also consistent with the data on the Ag^+^ ion release from the nanostructures (Figure 16). The thermal treatment of the nanoscale structures led to an increase in the amount of released silver ions by 4–6 times.

For nanorods and nanoplatelets, the heat treatment and an increase in the amount of Ag^+^ ions released contributed to an increase in antibacterial activity from 31% and 0% to 99% (Figure 15a, curves two and five, and curves three and six, respectively). For the flower-like nanoscale structures, the increase in the amount of the released Ag^+^ after the thermal treatment slightly increased their antibacterial activity (Figure 15a, curves one and four). The nanostructures subjected to the heat treatment showed a more effective reduction in the number of bacteria and reached 100% efficiency after 60 min of exposure, due to the migration of silver particles on the surface of the nanostructures, contributing to the direct contact of the noble metal with the bacterial membrane, reducing their size and leading to a more intense release of Ag^+^ ions.

**Figure 17:**
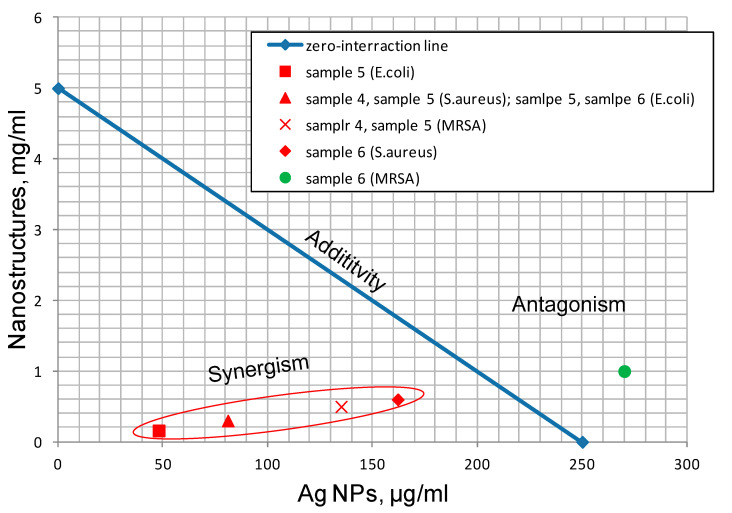
Isobologram of the combined action of γ-Al_2_O_3_ nanostructures and Ag nanoparticles. The content of silver was taken on the basis of 27 wt% metal content in the nanostructures.

The results obtained also allowed for estimating the nature of the mutual influence of the nanoscale structure components on their antibacterial activity. The MIC of the silver nanoparticles has been reported to range from 0.25 µg × mL^−1^ [54] to 550 µg × mL^−1^ [55], and depends on the method of nanoparticle production, their size and accompanying impurities. The wet, chemically synthesized silver nanoparticles [56] showed antimicrobial activity at a concentration of 250 µg × mL^−1^. Taking into account this value, isobolograms were plotted for the synthesized nanostructures (Figure 17) to evaluate the nature of the mutual influence of the components on the antibacterial activity of the samples. It can be seen that the synergistic effect with respect to *E. coli* and *S. aureus* was evident for all the nanoscale structures obtained with the thermal treatment (Figure 17). Against MRSA, a synergistic antimicrobial effect was also observed for the γ-Al_2_O_3_/Ag flower-like structures and γ-Al_2_O_3_/Ag nanorods. It should be noted that the synergistic effect for the γ-Al_2_O_3_/Ag flower-like nanoparticles with respect to *E. coli* would be maintained even if the silver MIC value was reduced to 50 µg/mL.

Thus, the results obtained indicated that the mechanism of bacteria inactivation through the synthesized nanostructures included both the adsorption factor and the action of Ag^+^ ions. According to the totality of the results obtained, one could conclude that the γ-Al_2_O_3_/Ag flower-like nanostructures had the highest antibacterial activity.

## 4. Conclusions

By varying the reaction conditions of the water oxidation of bimetallic Al/Ag nanoparticles, it is possible to obtain nanoscale structures with different morphologies, a positive ζ-potential, a developed surface and pronounced antimicrobial properties. The oxidation of the Al/Ag nanoparticles in water at 60 °C led to the formation of AlOOH flower-like nanoscale structures, inside which Ag nanoparticle agglomerates with an average size of 17 nm were concentrated. Under hydrothermal oxidation conditions, AlOOH nanoplatelets were formed with Ag nanoparticles, with an average size of 22 nm attached to their surface. In humid air, Al(OH)_3_ nanorods were formed, on the surface of which Ag nanoparticle agglomerates, with an average particle size of 19 nm, were fixed in the aluminum oxide phase. The thermal treatment led to the migration of silver nanoparticles to the periphery of the nanoscale structures, as well as to a decrease in the size of silver nanoparticles, which contributed to a significant increase in the antimicrobial activity of the samples, which was composed both due to the ability of nanostructures to adsorb bacteria and release Ag^+^ ions. Taking into account the Ag MIC value of 250 μg/mL, the nanoscale structures with flower-like and nanorod morphologies after the heat treatment were shown to demonstrate a synergistic antimicrobial effect against *E. coli*, *S. aureus* and MRSA, while nanoplatelets after the heat treatment demonstrated a synergistic antimicrobial effect against *E. coli* and *S. aureus*.

## Figures and Tables

**Table 1:** Conditions for obtaining nanocomposites.

Sample	Precursor	Synthesis Conditions				
		Time, Hours	Medium	Temperature, °C	Pressure, MPa	Relative Humidity, %
1	Al/Ag	1	Water	60	0.1	-
2	Al/Ag	72	Humid air	60	0.1	80
3	Al/Ag	6	Water	200	16	-
4	Sample 1	2	Air	500	0.1	-
5	Sample 2	2	Air	500	0.1	-
6	Sample 3	2	Air	500	0.1	-

**Table 2:** The MIC values (mg × mL^−1^) of the nanostructures against *E. coli*, *S. aureus* and *MRSA*.

Strain	MIC (mg × mL^−1^)					
	Sample 1	Sample 2	Sample 3	Sample 4	Sample 5	Sample 6
*E. coli* ATCC 25922	1.30	2.50	2.50	0.16	0.32	0.32
*S. aureus* ATCC 6538P	0.60	5.00	2.50	0.32	0.32	0.64
MRSA ATCC 43300	1.024	5.00	5.00	0.512	0.512	1.024

## Data Availability

Not applicable.
